# Physiological, Biochemical, and Growth Parameters of Fogera Cattle Calves to Heat Stress during Different Seasons in Sub-Humid Part of Ethiopia

**DOI:** 10.3390/ani11041062

**Published:** 2021-04-08

**Authors:** Michael Abera, Yesihak Yusuf Mummed, Mitiku Eshetu, Fabio Pilla, Zewdu Wondifraw

**Affiliations:** 1 Africa Center of Excellence for Climate Smart Agriculture and Biodiversity Conservation, Haramaya University, P.O. Box 138 Haramaya, Ethiopia; 2 Department of Animal Sciences, Debre Markos University, P.O. Box 269 Debre Markos, Ethiopia; zewduwondifraw@gmail.com; 3 School of Animal and Range Sciences, Haramaya University, P.O. Box 138 Haramaya, Ethiopia; yesihakyus@gmail.com (Y.Y.M.); eshetumit@yahoo.com (M.E.); 4 Department of Agriculture Environment and Food, University of Molise, Via Francesco De Sanctis s.n.c., 86100 Campobasso, Italy; pilla@unimol.it

**Keywords:** physiological, hematological, biochemical, growth, heat stress

## Abstract

**Simple Summary:**

Evaluation of Fogera cattle breed during the different seasons would help to determine their response to heat stress. This would assist in designing intervention strategies for the anticipated climate change. Therefore, this study aimed to determine physiological, hematological, biochemical, and growth parameters of Fogera cattle calves to heat stress in different seasons. We observed that the heart rate and respiration rate were increased by six beats per minute and four breaths per minute when the temperature-humidity index increased from 66 to 78, respectively. The positive relationship between the temperature–humidity index and physiological parameters further confirms that calves were good at thermoregulation at a temperature–humidity index value of 66. Thus, the temperature-humidity index value of 66 can be considered as optimum for high weight gain and normal physiological response to heat stress in Fogera cattle calves under their current production system. However, some more amelioration strategies such as better nutrition, availability of shade, and routine health management practice will further strengthen the resilience of the breed to heat stress in the future.

**Abstract:**

Fogera cattle are among indigenous breeds of cattle in the northern part of Ethiopia. However, their response to heat stress (HS) under different seasonal variations has not been well investigated. This study was aimed to determine physiological, hematological, biochemical, and growth parameters of Fogera cattle calves to HS during dry season, short rainy, and long rainy. A total of 72 calves (24 for each season) that were 6 months of age with an equal number of males and females were evaluated for physiological, hematological, biochemical, and growth parameters. Daily ambient temperature (AT) and relative humidity (RH) were recorded two times per day during the study periods from which the daily average temperature–humidity index (THI) was calculated. The study revealed higher AT and THI during dry and short seasons while higher RH was observed during the long rainy season. Physiological parameters except rectal temperature were affected by the seasons. Hematological parameters were also affected by season except for packed cell volume. Biochemical and growth parameters were also significantly affected by the seasons. THI was positively related with physiological but negatively with growth parameters. Thus, the THI value of 66 can be considered as optimum for high weight gain and normal physiological response to HS in Fogera cattle calves under their current production system.

## 1. Introduction

Cattle are generally the livestock species most susceptible to water and nutritional stresses engendered by climate change [1]. High ambient temperature (AT) in combination with relative humidity (RH) compromises the animals’ ability to lose heat to the surroundings resulting in heat stress (HS). HS lowers the feed intake of an animal, which reduces their productivity in terms of milk yield, body weight, and reproductive performance. Besides, HS exerts a negative effect on the dry matter intake (DMI) and growth performance of calves and heifers. It was reported that dairy calves born in summer tended to have a lower average daily gain (ADG) than those born in winter [2].As calves consume agiven volume of milk or milk replacer and starter ad libitum daily, the main effect of HS on DMI for calves might lie in the starter [3]. Baccari et al. [4] reported that lower feed intake, ADG, and feed efficiency of Holstein heifers under HS conditions (32.5–34 °C environment) compared with cooler conditions (18–20 °C environment). Nonaka et al. [5] also found that daily dry matter intake and ADG of prepubertal Holstein heifers at 33 °C environment dropped by 9% and 22%, respectively, compared to those raised at 28 °C environment. However, water intake increased by 23% due to additional evaporative water loss, such as sweating. Neuwirth et al. [6] reported that bull calves responded with higher heart rate (HR), arterial pressure, skin temperature, plasma cortisol, and thyroxine concentrations to acute HS only above 32.2 °C at 60% relative humidity, which corresponds to a THI of 80.6.

The temperature–humidity index (THI) is the widely used index to measure the magnitude of HS in animals [7]. However, some studies show that THI values only serve as a rough measure of HS effect on production [8]; they call for necessary adjustments because the environmental stimulus includes other factors such as wind speed and solar radiation [9]. Moreover, the THI threshold for calves and heifers remains unknown because of the very limited information available related to THI and HS on calves. Therefore, more studies will help to quantify THI for calves and even explore new indices to indicate the level of HS [3]. Under HS, the animals exhibit various behavioral, physiological, hematological, biochemicals, and endocrine adjustments to reduce stress inflicted. HS can be assessed by changes in physiological, hematological, and biochemical responses. These are the values that help in determining the adaptation of the animal to the existing environment [10,11,12]. Metekel ranch is located in northwestern Ethiopia characterized as a sub humid environment, meaning very wet and dry seasons [13]. Thus, the young stock population existing in this ranch was expected to undergo HS, particularly during the dry season. Therefore, the hypothesis of the proposed study was based on the assumption that seasonal changes in AT and RH induce heat stress in animals, which may be reflected by alteration in physiological, hematological, biochemical, and growth responses.

Fogera cattle breed is among the indigenous breeds of cattle in the northern part of Ethiopia and has been raised at Metekel ranch since 1986 for breed improvement and conservation. Tesfa et al. [14] reported that the current population size of the breed is about 55,646. However, it is declining through time due to its wide area distribution in the region and genetic admixture. The same author also reported that the overall birth and weaning weight of Fogera cattle breed at Andassa livestock research center (ALRC) was 21.4 ± 0.09 kg and 102.2 ± 0.77 kg, respectively. Similar to livestock agriculture in our world, Fogera cattle breed is expected to face climate change and variability in the future. Hence, it is essential to evaluate HS effect on the breed, particularly, as young stock calves are more susceptible to HS than adult ones. The study showed that the thermo neutral zone (TNZ) of a 1-month-old calf is between 13 and 25 °C and the TNZ of a heifer with 0.8 kg daily gain is between 0 and 15 °C [15].Moreover, the mean rectal temperature(RT), respiration, pulse, and heart rate (per minute) were significantly higher in young animals than in adult cows and bulls [16]. However, the response of the Fogera cattle calves to the HS under different seasonal variations has not been well investigated. Therefore, this study was aimed to determine the degree of physiological, hematological, biochemical, and growth responses of Fogera cattle calves during different seasons.

## 2. Materials and Methods

### 2.1. Description of the Study Area

The study was carried out in Metekel ranch, located in Guangua district of Awi zone in Amhara National Regional State and is situated at about 505 km North-west of Addis Ababa, 200 km from the regional town Bahir Dar. Its altitude ranges from 1500 to 1680 m above sea level (m.a.s.l). Metekel ranch is located at 10°57’6.5232” N latitude and 36°30’45.0864” E longitude.Theranch was established in 1986 for the Fogera cattle conservation and improvement program. The vegetation is mostly composed of perennial and annual grasses and a few scattered trees. According to Melak [17], *Cynodondactylon, Digitariaternata, Digitariavelutina, Brachiariadictyoneura, Commelinagenghalensis*, and *Panicum maximum* were the most palatable grass species while *Schinusmolle, Casuarina equisetifolia*, and *Eucaulyptus camaldulensis* were the predominant tree species. The annual mean RH is 61.7% and it reaches peak from June to October (76.7–83.8%). The ranch receives an average annual rainfall of 1730 mm and the average temperature ranges from 13.7 to 29.5 °C [18].Rainfall distribution is bimodal. According to Ababa [19], the study area has three seasons classified as the dry season (October–January), short rainy season (February–May), and long rainy season (June–September).

### 2.2. Experimental Design and Animal Management

A total of 72 calves, 6 months of age were randomly selected and used in this study. The calves were grouped into three of 24 animals with an equal number of males and females. Then, they were subjected to three different seasons such as dry season (January), short rainy season (April), and the long rainy season (July) for 30 days each. These months were selected since they are assumed to be representatives of the three seasons in the study area. The calves were fed as per the National Research Council [20] feeding standards with concentrate supplement in the simple shade. Additionally, they were also allowed to graze from 09:30 to 12:30 hours throughout the study period [21]. About 55, 26, 17, 1, and 1% maize, nougseedcake, wheat bran, salt, and ruminant premixes (minerals and vitamins) mixtures, respectively, were used as concentrate supplements. Before the study’s commencement, calves were treated with Ivermectin (Hebei Hope Harmony Pharmaceutical Co., Ltd., Hebei Province, China) and Albendazole (Ashish life science Pvt., Ltd., Maharashtra, India) for external and internal parasite treatments, respectively. All animal handling practices were followed the international guiding principles listed by the council for international organizations of medical sciences and the international council for laboratory animal science [22]. Meteorological parameters such as AT (°C) and RH (%) were collected two times per day, during morning and afternoon to get maximum and minimum values inall the experimental periods using a Hygrothermometer. Then, average daily THI was calculated from AT and RH using the equation; THI = (1.8 × AT + 32) − [(0.55 − 0.0055 × RH) × (1.8 × AT − 26)] developed by the National Research Council [20] for ruminant animals. Where, THI= temperature–humidity index, AT = ambient temperature (°C), and RH = relative humidity (%).This equation was used for calves in this study because the THI threshold for calves and heifers remains unknown due to minimal information about THI and HS [3].For the same reason, the classification reported by Habeeb et al. [23] was also adopted to quantify the intensity of HS in calves. Thus, calves were assumed to be in comfort zone if (THI < 68), mild discomfort zone if (68 < THI < 72), discomfort if (72 < THI <75), alert (75 < THI < 79), danger (79 < THI < 84), and emergency (THI > 84).

### 2.3. Data Collection

#### 2.3.1. Physiological Parameters

Rectal temperature (RT) was measured using a clinical veterinary thermometer inserted at the animal’s rectum wall at a depth of approximately 3.0 inch for 3 min. Heart rate (HR) expressed in the number of beats per minute and respiratory rate (RR) expressed in breaths per minute were measured using a stethoscope and a stopwatch for the 30 s and multiplying the results by two to obtain the counts per minute.

#### 2.3.2. Blood Sample Collection and Analysis

In each season, three times, blood samples were collected for each hematological and biochemical analysis on days 1, 15, and 30 following the procedure of Nikhil et al. [21]. A total of 432 blood samples were collected from the jugular vein of calves. Out of these, about 216 blood samples were collected in tubes containing ethylene diamine tetraacetic acid (EDTA)as an anticoagulant for hematological analysis while 216 blood samples were collected in serum separator tubes(SST)for biochemical analysis. Hematological study was performed within 24 h after blood collection. Whole blood was collected between 10:00 and 11:00 h. Then, the collected blood sample was analyzed by Hematology analyzer Germany version 2.5 to determine the value of white blood cell (WBC), red blood cell (RBC), hemoglobin (HGB), packed cell volume (PCV), mean corpuscular volume (MCV), mean corpuscular hemoglobin (MCH), and mean corpuscular hemoglobin concentration (MCHC).The plasma was separated from 216 blood samples by centrifugation at 3500 rpm for 10 min at 24 °C and stored at 4 °C for estimation of biochemical parameters until further analysis. Moreover, the blood serum analysis method is shown in Table 1.

#### 2.3.3. Growth Parameters

The calves were weighed at the beginning of the study and every 15 days thereafter using the weighing balance in each season for 30 days. All measurements were taken after overnight withdrawal of feed and water. Total gain (TG) was calculated as the difference between final body weight (FBW) and initial body weight (IBW). Then, average daily gain (ADG) was determined by dividing the differences of the FBW and IBW by the number of experimental periods (30 days for each season).

### 2.4. Statistical Analysis

The average of days 1, 15, and 30 in each season for all parameters were obtained and comparisons were made among seasons [21]. The data obtained on various parameters were statistically analyzed using a two-way analysis of variance. The data were analyzed by the general linear model (PROC GLM) using SAS software version 9.4. Sex of the calves and season were fitted as independent variables while physiological, hematological, biochemical, and growth parameters were fitted as response variables. When the GLM showed the presence of a significant difference between the parameters, the Tukey Kramer test was used for mean comparison. Moreover, the Pearson correlation test was performed to check the strength of the relationship between physiological parameters and THI as well as growth parameters and THI.

The general linear model used for the analysis of physiological parameters was

(1) Y=μ+Si+Kj+(S × K)ij+eijk

where; *Y_ijk_* = the response variables (HR, RR, RT); *µ* = overall mean; *S_i_* = effect of sex (female, male); *k_j_* = effect of season (dry season, short rainy season, long rainy season); (*S* × *K*)*_ij_* = interaction between sex and season; *e_ijk_* = random error.

The general linear model used for the analysis of hematological parameters was

(2) Y=μ+Si+Kj+(S × K)ij+eijk

where; *Y_ijk_* = the response variables (WBC, RBC, HGB, PCV, MCV, MCH, MCHC); *µ* = overall mean; *S_i_* = effect of sex (female, male); *k_j_* = effect of season (dry season, short rainy season, long rainy season); (*S* × *K*)*_ij_* = interaction between sex and season; *e_ijk_* = random error.

The general linear model used for the analysis of biochemical parameters was

(3) Y=μ+Si+Kj+(S × K)ij+eijk

where; *Y_ijk_* = the response variables (total protein, urea, creatine, glucose, total cholesterol); *µ* = overall mean; *S_i_*= effect of sex (female, male); *k_j_* = effect of season (dry season, short rainy season, long rainy season); (*S* × *K*)*_ij_* = interaction between sex and season; *e_ijk_* = random error.

The general linear model used for the analysis of growth parameters was

(4) Y=μ+Si+Kj+(S × K)ij+eijk

where; *Y_ijk_* = the response variables (IBW, FBW, TG, ADG); *µ* = overall mean; *S_i_* = effect of sex (female, male); *k_j_* = effect of season (dry season, short rainy season, long rainy season); (*S* × *K*)*_ij_*= interaction between sex and season; *e_ijk_* = random error.

## 3. Results

### 3.1. Meteorological Variables during the Study Periods

Higher daily mean AT was observed during the dry and short rainy seasons compared to the long rainy season (Table 2) (*p* < 0.05). Moreover, a higher mean daily THI was noticed during the dry and short rainy seasons (*p* < 0.05). Whereas, higher mean daily RH was observed during the long rainy season (*p* < 0.05). The maximum AT in dry and short rainy seasons was greater than the long rainy seasons. However, there was no statistically significant difference among the three seasons in minimum AT during the experimental periods. On the other hand, maximum RH increased by 40.95 and 47% during the long rainy season over the dry and short rainy seasons, respectively. Moreover, the minimum RH increased by 49.18 and 51.71% during the long rainy season over the dry and short rainy seasons, respectively.

### 3.2. Physiological Parameters

Physiological parameters of calves were significantly affected by season except for RT (Table 3). HR was increased by 5.7 and 4.1 beats per minute during the dry season over long and short rainy seasons, respectively. Moreover, RR was increased by 3.8 and 3.4 breaths per minute during the dry season over the long and short rainy seasons, respectively. The calves’ HR was significantly affected by sex (Table 3) (*p* = 0.007). Moreover, RR of the calves were significantly affected by the sex of the calves (*p* = 0.046). Male calves had consistently higher HR and RR than female calves. However, RT of the calves were not significantly affected by the sex of the calves (*p* = 0.165). On the other hand, physiological parameters were affected by season and sex interaction effect except for RT of the calves.

### 3.3. Hematological Parameters

**Figure 1:**
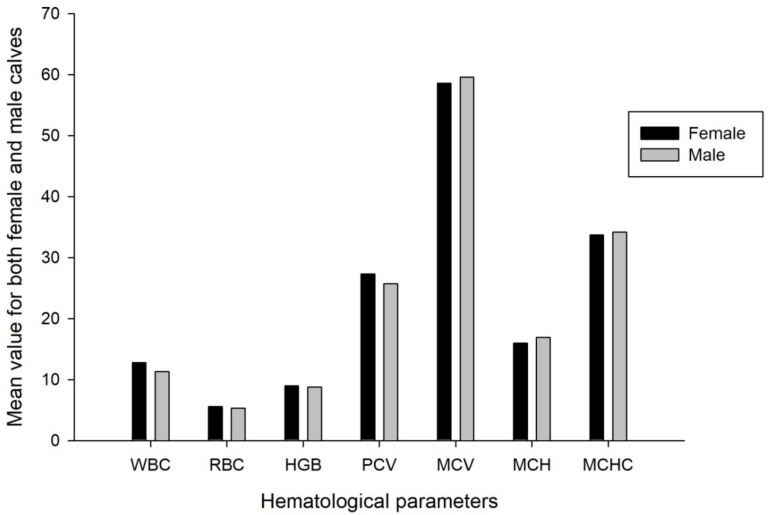
Effect of sex on hematological parameters. WBC (×10 µL^−3^) = white blood cell in micro liter, RBC (×10 µL^−6^) = red blood cell in micro liter, HGB (g dL^−1^) = hemoglobin in gram per deciliter, PCV (%) = packed cell volume in percentage, MCV (fL) = mean corpuscular volume in femtoliter, MCHC (g dL^−1^) = mean corpuscular hemoglobin in gram per deciliter, MCHC (g dL^−1^) = mean corpuscular hemoglobin concentration in gram per deciliter.

Hematological parameters were significantly affected by season variation except for PCV (Table 4). A significantly lower WBC value was observed in the dry season compared to short and long rainy seasons (*p* = 0.011). Moreover, considerably lower values of HGB were observed in the dry and long rainy seasons than short rainy seasons (*p* = 0.000). Appreciably, the concentration of RBC was higher in the short rainy season followed by dry and long rainy seasons, respectively (*p* = 0.000).The concentration of MCV was significantly affected by the variation in season (*p* = 0.000). Lower mean MCV was observed in the short rainy season compared to dry and long rainy seasons. MCH was significantly lower during the short rainy season than dry and long rainy seasons. Considerably, lower MCHC values were observed in the dry season than short and long rainy seasons (*p* = 0.000). The interaction effect of sex and season did not affect all hematological parameters (Table 4). Almost all of the hematological parameters were not significantly affected by the sex of the calves except PCV (Table 4) (Figure 1).It was noticed that higher PCV was observed in females than male calves (Figure 1) (*p* = 0.028).

### 3.4. Biochemical Parameters

The least-square mean value of total protein, cholesterol, and glucose concentration was lower in the dry season than short and long rainy seasons (Table 5) (*p* = 0.000). Conversely, creatine, and urea concentration were higher in dry and short rainy seasons compared to the long rainy season (*p* = 0.000). Our result indicated that most of the biochemical parameters were not affected by sex and season interaction effect except for creatine, and urea (Table 5). Significantly, female calves had higher creatine during the short rainy season than males. However, there was no significant difference in creatine during the dry and long rainy seasons. Female calves had higher blood urea than males in the short rainy seasons, but male calves had higher blood urea than females during long rainy seasons.

### 3.5. Growth Parameters

All growth parameters considered in our study were significantly affected by the seasons (Table 6). In the long and short rainy seasons, IBW was increased by 15.8 and 14.5 kg compared to the dry season, respectively. Moreover, in the long and short rainy seasons, the FBW was increased by 22 and 10.6 kg compared to the dry season, respectively. Total gain (TG) was increased by 10.3 and 4 kg in the long and dry seasons, respectively, over the short rainy season. Similarly, ADG was increased by 300 and 100 g/day in the long and dry seasons, respectively, over the short rainy season. All growth parameters were not significantly affected by the sex of the calves. However, TG and ADG were significantly affected by sex and season interaction. TG of female calves was higher in dry and short rainy seasons whereas male calves were 9 kg higher than females in the long rainy season. Moreover, significantly higher ADG was observed in females in the dry and short rainy seasons while higher ADG was observed in males in the long rainy season (Table 6).

### 3.6. Relationship between THI and Physiological Parameters

**Figure 2:**
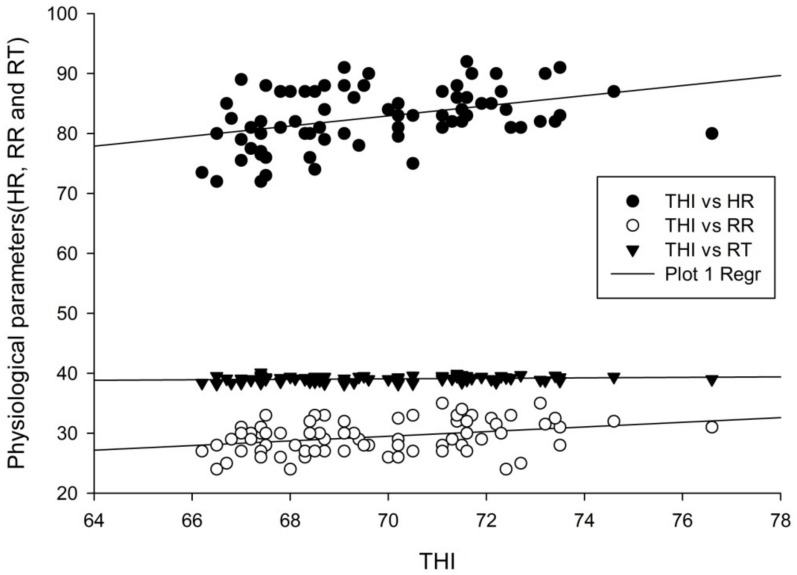
Relationship between the temperature–humidity index and physiological parameters of calves.THI, HR, RR, and RT (°C) are temperature–humidity index, heart rate (beats per minute), respiration rate (breaths per minute), and rectal temperature in degree centigrade, respectively.

The study revealed that THI was positively correlated with HR and RR. The HR increased by six beats per minute and RR increased by four breaths per minute when the THI increased from 66 to 78 (Figure 2). Moreover, the Pearson correlation test showed that there was a relationship between THI and HR (r = 0.395, *p* = 0.001) and THI and RR (r = 0.331, *p* = 0.005). The result also showed a positive relationship between HR and RR as illustrated in Figure 2. Thus, from this study, it can be noticed that the THI value of 66 is a critical value for all physiological parameters considered in this particular breed under the current situation. Up to this critical value, animals could perform well since there was a minimal effect of HS. However, as THI advances calves may lose their performance to maintain their constant body temperature.

### 3.7. Relationship between THI and Growth Parameters

**Figure 3:**
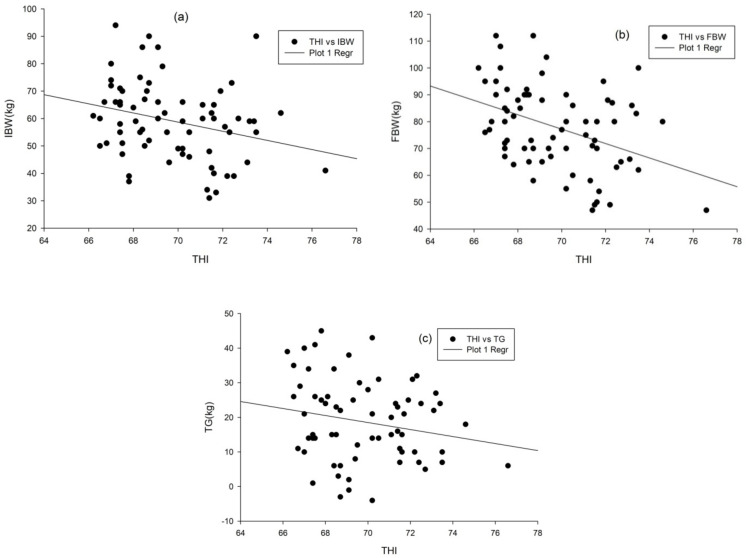
Relationship between THI and IBW (**a**), THI and FBW (**b**), and THI and TG (**c**) THI, IBW, FBW, and TG, are temperature–humidity index, initial body weight, final body weight, and total gain, respectively.

Growth parameters such as IBW, FBW, and TG were negatively associated with the increase in THI (Figure 3). The Pearson correlation test also confirmed that there was a negative relationship between THI and IBW (r = −0.274, *p* = 0.02); THI and FBW (r = −0.397, *p* = 0.001) (Figure 3a,b). Furthermore, there was a negative relationship between THI and TG (r = −0.205, *p* = 0.042) as indicated in Figure 3c. From Figure 3a–c, it can be generalized that all the growth parameters were declining after the THI value of 66. However, up to this value, all the growth parameters were slightly increasing. This result further justifies that THI plays a significant role in determining young calves’ performance, especially during dry and short rainy seasons. Thus, greater attention should be given to environmental factors such as AT and RH when designing any management interventions for improving their performance.

## 4. Discussions

### 4.1. Meteorological Variables during the Study Periods

The AT and RH were the commonly used measure of HS in animals until THI was derived [21,24]. THI accounts for AT and RH’s combined effect and is considered one of the best methods to evaluate HS in animals [25]. However, previous studies showed that THI values only serve as a rough measure of HS effect on production [8]; they call for necessary adjustments because the environmental stimulus includes other factors such as wind speed and solar radiation [9]. The result showed that AT values were higher than the critical values reported by Hahn [15] for TNZ for a heifer with 0.8 kg daily gain in all the seasons while AT values were within TNZfor1-month-old calves reported by the same author during the long rainy season. Conversely, RH values in dry and short rainy seasons were lower than the critical values reported by Neuwirth et al. [6] for bull calves whereas higher than the report of Neuwirth et al. [6] during the long rainy season. Based on the assumption by Habeeb et al. [23] calves were in a discomfort zone during the dry and short rainy seasons while in mild discomfort during the long rainy season. However, the THI value was relatively lower during all seasons than reported by Neuwirth et al. [6] for bull calves. This difference might be due to breed, location, and management practices. Therefore, it can be concluded that under the current situation the overall meteorological data indicated that the animals were heat-stressed in all three seasons as confirmed by upper critical AT, RH, and THI. However, the impact of HS was high in the dry season and short rainy seasons compared to the long rainy season evidenced by eminent AT and THI in the dry season and short rainy season compared to the long rainy season. This discomfort zone for calves observed during the dry season and short rainy seasons in this study indicated the need to implement further amelioration strategies in the anticipated climate change in the future.

### 4.2. Physiological Parameters

No difference in RT under different seasons indicates a better adaptive capacity of Fogera cattle calves to their current production environment under different conditions. The HR of the calves was significantly affected by seasons (*p* = 0.0001). This might be because there was high AT and THI during the dry season compared to both short and long rainy seasons. The mean RR noticed in the dry season was comparable with Hariana breed’s value during the summer [26]. Moreover, the mean RR observed in the short and long rainy seasons was higher than the value reported for Hariana, and Sahiwal breeds, respectively [26]. Thus, it can be suggested that Fogera cattle calves are good at heat tolerance under tropical climate of THI less than or equal to 66.

An increase in RR was able to eliminate excess heat and maintain a constant deep body temperature [27]. It is generally true that in matured animals due to physiological differences, males are better at thermoregulation than females. Likewise, our finding also confirmed that higher HR and RR were observed in male calves than females, showing that male calves are relatively better at heat tolerance than females. These differences might be emanated from increased HR and RR in male calves at elevated environmental AT and RH. Neuwirth et al. [6] also reported that bull calves responded with HR, arterial pressure, skin temperature, plasma cortisol, and thyroxine concentrations to acute HS only above 32.2 °C at 60% RH which is consistent with our findings. In contrary to our findings, it has been reported that heifers experience an increased heart rate during HS [28,29].This helps maintain blood pressure as a response to the elevated vasodilatation and increased blood flow caused by HS [28]. Another study also showed that heifers were reported to deposit more fat than steers [30], which might affect their thermoregulation at higher AT, RH and THI than young bulls.

### 4.3. Hematological Parameters

Significantly higher PCV values were noticed in females than male calves. The difference in PCV between females and male calves in this study might be due to sex differences which are further linked with differences in cortisol hormone production in response to HS. Contrary to this result, Kubkomawa et al. [31] reported males having higher mean PCV than female cattle.

However, there was no significant difference in WBC, RBC, HGB, MCV, MCH, and MCHC between male and female calves. According to Celik et al. [32], HGB and MCH levels were not statistically different between the sex of the calves, which is concordant with our findings. Moreover, Camargo et al. [33] also did not find a significant difference between males and females in MCHC, which is also in line with our result.

The lower WBC counts noticed in the dry season might be due to the body system’s response to stress stimuli compared to the long rainy season. During the study periods, irrespective of the seasons, calves were given similar routine health management such as deworming and continuous follow-up of the health condition. Moreover, they were given feed with good nutrient content in addition to shade from the extreme sunlight. This suggested that more amelioration strategies need to be devised for calves to face severe climate change anticipated in the future. Likewise, Mirzadeh et al. [34] conducted a study and reported lower WBC in summer as compared to spring, autumn, and winter seasons in all age groups of Iranian cattle which is in line with our result.

The mean value of total RBC was significantly lower in the long rainy season than short and dry seasons (*p* = 0.000). Moreover, Naik et al. [16] did a similar study and found significantly higher RBC values in summer compared to winter and monsoon in different age groups in Punganur cattle. Aengwanich et al. [35] did not find any significant changes in RBC among summer, rainy, and winter seasons in beef cattle. The higher number of RBC in dry and short rainy seasons might be associated with relatively higher stress in the body, requiring more oxygen transport throughout the body. Furthermore, the lower RBC during the long rainy season could be due to increased water intake through the lush grasses that were available in that season [36] or high RH observed in the long rainy as compared to other seasons would have compromised evaporative heat loss mechanisms resulting in HS and, therefore, animals would have ingested more water and subsequent hemodilution and hence decreased RBC [37].

The lower HGB observed in the dry and long rainy seasons may be because of smaller RBC counts observed in these seasons (Table 4).The lower MCV and MCH in the short rainy season and MCHC in the dry season in this study might be due to a low amount of HGB present per RBC. However, the effect of season was not significantly affected PCV (*p* > 0.05). Similar findings were reported by Aengwanichet al. [35] who reported that season did not affect hematological parameters of heifers in the northeastern part of Thailand. Contrary to this finding, significantly lower PCV was noticed in summer as compared to winter in all age groups of cattle [34]. Despite upper critical values of AT, RH, and THI in all three seasons and the absence of variation in PCV indicates once again the adaptive potential of Fogera cattle calves to their current production environment.

### 4.4. Biochemical Parameters

The lower total protein observed in this study might be due to the higher AT and THI observed in the dry season. Similarly, Dar et al. [38] reported significantly lower serum protein levels during the summer season in >1 year of age than in the winter season in Badri cattle. Contrary to this result, Nikhil et al. [21] did not find a significant change in total protein concentration among summer, rainy, and winter seasons in crossbred female calves of 6–12 months age in hot and humid tropics. Our study showed that total cholesterol concentration was higher in the long rainy season followed by short rainy and dry seasons (*p* = 0.000). Farooq et al. [39] also showed significantly lower cholesterol during the hot season compared to cool dry winter in adult Cholistani bulls. Considerably, low plasma cholesterol concentration during dry, and short rainy seasons may be due to HS-induced accelerated fat catabolism [40] or increased lipid mobilization by peripheral tissues [41] or may be due to reduced liver capacity [42] under HS in these seasons.

The decreased glucose concentration in dry and short rainy seasons compared to long rainy seasons could be due to more energy requirement to the animal to disperse more heat to maintain the body temperature at a normal level or could be due to heat stress-induced depressed dry matter intake [43]. Similarly, Nikhil et al. [21] also reported significantly lower glucose in pre-monsoon followed by monsoon and post-monsoon in crossbred female calves in hot and humid tropics. The higher serum creatine concentration during dry and short rainy seasons might be because of excess muscular catabolism for energy supply as voluntary feed intake is reduced due to these seasons [39]. The serum creatine concentration was smaller than values reported for Gir, Sahiwal, Kangayam, and Tharparkar but higher than Rathi cattle. However, the result is comparable with DeoniZebu cattle breeds of India during early summer [44]. A higher concentration of creatine during summer stress was also reported by Dar et al. [38].

Blood urea concentration was significantly higher during the dry season, followed by short and long rainy seasons (Table 5). Likewise, a higher blood urea concentration was reported during summer compared to winter in >1 year age of Badri cattle [38]. Our results were also in agreement with Rasouliet al. [11], who reported higher blood urea nitrogen during the summer season. This increase may be due to the utilization of amino acids for energy. The other reason may be due to protein mobilization from muscle tissue and stress-related cortisol elevation, which increases catabolism of body proteins [38].

### 4.5. Growth Parameters

The increase in IBW and FBW of calves in the long and short rainy season was attributed to natural pasture availability and decrease in maximum temperature compared to the dry season. The increment in IBW and FBW of calves further attributed to the increase in TG by 10.3 kg and ADG 300 g/day in the long rainy season as compared to dry and short rainy seasons. Despite the change in IBW and FBW in different seasons in the current study, the result was slightly smaller than the value reported by Tesfa et al. [14] in the dry and long rainy seasons, respectively, for the same breed at ALRC. This difference might be due to the difference in availability of forage at these locations and environmental temperature which further inhibits feed intake and growth of calves at Metekel ranch as it is hotter than ALRC.A previous study conducted by Pereira et al. [45] also showed that feed intake was reduced by 11.4% in Limousine calves under thermal stressful conditions, consistent with our finding. However, the ADG in this result was slightly higher than reported for the same breed in dry and long rainy seasons [14], respectively. The difference could be because of the short time experiment and the small number of observations used in this study. The increase in TG and ADG in females over the males during dry and short rainy seasons may be due to female calves’ special care rendered to them as a replacement and distribution to the surrounding farmers. However, all growth parameters were not significantly affected by sex differences (Table 6). The result is in line with Mekuriaw et al. [46] for Ogaden breed and [14,47] for Fogera cattle breed.

### 4.6. Relationship between THI and Physiological Parameters

As THI increased from 66 to 78, the HR of the calves was increasing from 80 to 90 beats per minute (Figure 2) (*p* < 0.05). Moreover, there was an increase in RR of the calves as THI advances from 66 to 78. This might be due to the increase in THI affecting internal physiological activities such as the calves’ metabolic rate, further increasing the HR and RR to respond against HS. The current result is in line with the findings of Aziz et al. [24] who reported that THI exhibited a positive correlation with the respiratory rate and pulse rate in crossbred cattle calves in Indian. Constant RT and increased HR and RR when calves are exposed to a THI from 66 to 78, indicated that the calves had a high heat tolerance level. They maintained thermal balance by removing excess heat through enhanced respiratory rate and pulse rate [24]. Moreover, the positive relationship between physiological parameters (HR and RR) justifies that calves were well adaptive to their production environment by maintaining their homeostasis through elevated HR and RR. Bianca [48] also found a positive correlation between HR and RR at severe HS in Ayrshire bull calves.

### 4.7. Relationship between THI and Growth Parameters

The decline in the growth parameters might be because calves dissipate heat from their body to adapt to the increase in THI, especially during the dry season. Moreover, the decrease in growth rate with the increase in THI might be because animals used most of the energy to maintain their homeothermy instead of growth [24]. Therefore, to maintain the growth rate and sustain this breed’s adaptability under increased THI, appropriate feeding, shade provision, and frequent watering may be suggested for the coming climate change. A similar study conducted in Japanese black calves indicated that body weight gain with THI of >75 was significantly lower than that with THI ranging from 56 to 60 [49]. Moreover, Broucek et al. [50] showed that calves under HS conditions (74.8 of THI) had reduced starter intake compared with those raised under moderate conditions (59.7 of THI).Colditz and Kellaway [51] also reported that heifers raised under HS condition (38 °C environment) had reduced feed intake and ADG compared to those maintained under cool ambient conditions (17 °C environment) which is corroborated with this result.

## 5. Conclusions and Recommendations

Our study revealed that the existence of upper critical AT, RH, and THI in all three seasons indicated the HS reality in all the animals in the study area. There was a change in growth parameters such as IBW, FBW, TG, ADG, but no change in physiological (RT) and hematological (PCV) parameters with varying seasons. From the finding, it is possible to say animals adapted to the existing stress by similar physiological and hematological responses. There was also a positive relationship between THI and physiological parameters. This implies that as THI gets advanced, Fogera cattle calves adjust their body temperature by increasing their HR and RR. Thus, Fogera cattle calves are good at thermoregulation under the tropical climate of THI less or equal to 66. Therefore, the THI value of 66 can be considered optimum for high weight gain and normal physiological responses to HS in Fogera cattle calves under their current production system. However, some more amelioration strategies such as better nutrition, availability of shade, and implementation of routine health management practice should be considered for the resilience of the breed to HS in the future. Future research should be done in a similar environment by including different indigenous breeds to select the best-performing animals at higher THI values.

## Figures and Tables

**Table 1:** Serum sample and analysis method.

Parameters	Method of Analysis	Instrument Used
Total protein	Biuret method	A25 biochemistry analyzer (Wreck and Mended enterprise, Thane West, Thane, Maharashtra, India) by using the reagents developed for each parameter test
Urea	An enzymatic method (GLDH method)	
Creatine	Photometric colorimetric test for kinetic measurements (Jaffe’-reaction)	
Glucose	An enzymatic colorimetric test (GOD-PAP method)	
Total cholesterol	An enzymatic colorimetric method with lipid clearing factor (CHOD-PAP method)	

**Table 2:** Daily meteorological variables during the study period (mean ± SE).

Meteorological Variables		DS (January)	SR (April)	LR (July)
Ambient temperature (°C)	Maximum	33.1 ± 0.5 ^a^	32.9 ± 0.6 ^a^	23.1 ± 0.3 ^b^
	Minimum	21.3 ± 0.6 ^a^	21.0 ± 0.7 ^a^	20.7 ± 0.2 ^a^
	Mean	27.0 ± 0.4 ^b^	26.9 ± 0.4 ^b^	21.8 ± 0.2 ^a^
Relative humidity (%)	Maximum	47.6 ± 1.1 ^a^	41.5 ± 1.9 ^b^	88.6 ± 0.6 ^c^
	Minimum	30.9 ± 0.8 ^a^	28.3 ± 0.8 ^a^	80.0 ± 1.2 ^b^
	Mean	39.2 ± 0.8 ^a^	34.9 ± 1.2 ^b^	84.3 ± 0.8 ^c^
THI	Maximum	81.7 ± 0.6 ^a^	80.7 ± 0.9 ^a^	72.5 ± 0.6 ^b^
	Minimum	65.6 ± 0.7 ^a^	65.0 ± 0.8 ^b^	67.9 ± 0.2 ^b^
	Mean	72.7 ± 0.5 ^a^	72.0 ± 0.4 ^a^	69.6 ± 0.3 ^b^

^a–c^ Rows with different superscripts for same parameter are significantly different at *p* < 0.05, THI = temperature–humidity index, DS = dry season, SR = short rainy season, LR = long rainy season.

**Table 3:** Effects of season on physiological parameters of male and female calves (mean ± SE).

Parameters	DS	Season		Sex		*p*-Value		
		SR	LR	Female	Male	Season	Sex	Season × Sex
HR	86.0 ± 0.7 ^a^	81.9 ± 0.8 ^ab^	80.3 ± 1.1 ^b^	81.5 ± 0.9 ^b^	84.0 ± 0.7 ^a^	0.0001	0.0070	0.0030
RR	31.8 ± 0.4 ^a^	28.4 ± 0.5 ^b^	28.0 ± 0.4 ^b^	28.9 ± 0.5 ^b^	29.9 ± 0.4 ^a^	0.0001	0.0460	0.0470
RT	39.1 ± 0.1	39.0 ± 0.1	38.9 ± 0.09	39.1 ± 0.1	39.0 ± 0.1	0.2680	0.1650	0.9450

^a,b^ Rows with different superscripts for same parameter are significantly different, DS = dry season, SR = short rainy season, LR = long rainy season, HR = heart rate (beats per minute), RR = respiration rate (breaths per minute), RT (°C) = rectal temperature (degree centigrade).

**Table 4:** Effect of season on hematological parameters (mean ± SE).

Parameters	DS	Seasons		Sex		*p*-Value		
		SR	LR	Female	Male	Season	Sex	Season × Sex
WBC(×10 µL^−3^)	10.3 ± 0.6	12.2 ± 0.7	13.6 ± 1.0	10.2 ± 0.7	10.5 ± 1.0	0.011	0.106	0.211
RBC(×10 µL^−6^)	5.2 ± 0.3	8.3 ± 0.3	2.9 ± 0.1	5.2 ± 0.2	5.3 ± 0.5	0.000	0.290	0.318
HGB (g dL^−1^)	8.0 ± 0.2	10.1 ± 0.2	8.5 ± 0.3	8.0 ± 0.3	8.1 ± 0.2	0.000	0.333	0.378
PCV (%)	26.3 ± 0.5	27.3 ± 0.8	26.0 ± 0.4	27.2 ± 0.8	25.3 ± 0.6	0.275	0.028	0.609
MCV (fL)	50.5 ± 1.2	33.7 ± 0.9	92.8 ± 0.8	51.4 ± 1.3	49.5 ± 2.1	0.000	0.275	0.084
MCH (pg)	15.7 ± 0.6	12.8 ± 0.9	31.1 ± 1.1	15.7 ± 1.0	15.7 ± 0.8	0.000	0.622	0.615
MCHC (g dL^−1^)	31.1 ± 0.9	37.5 ± 0.5	33.2 ± 1.1	30.6 ± 1.5	31.6 ± 0.9	0.000	0.614	0.135

DS = dry season, SR = short rainy season, LR = long rainy season, WBC (×10 µL^−3^) = white blood cell in micro liter, RBC(×10 µL^−6^) = red blood cell in micro liter, HGB (g dL^−1^) = hemoglobin in gram per deciliter, PCV (%) = packed cell volume in percentage, MCV (fL) = mean corpuscular volume in femtoliter, MCHC (g dL^−1^) = mean corpuscular hemoglobin in gram per deciliter, MCHC(g dL^−1^)= mean corpuscular hemoglobin concentration in gram per deciliter.

**Table 5:** Biochemical parameters for Fogera cattle calves as affected by seasons (mean ± SE).

Parameters	Seasons			Sex		*p*		
	DS	SR	LR	F	M	Season	Sex	Season × Sex
Total protein (g dL^−1^)	6.6 ± 0.1 ^a^	7.9 ± 0.2 ^b^	8.0 ± 0.3 ^b^	7.5 ± 0.2	7.5 ± 0.2	0.000	0.728	0.107
Total chol. (mg dL^−1^)	96.9 ± 1.1 ^a^	98.7 ± 1.4 ^a^	103.7 ± 1.1 ^b^	100.9 ± 1.0	98.7 ± 1.1	0.000	0.098	0.29
Glucose (mg dL^−1^)	70.4 ± 0.9 ^c^	84.3 ± 0.9 ^b^	90.2 ± 1.0 ^a^	81.4 ± 1.6	81.8 ± 1.6	0.000	0.731	0.073
Creatine (mg dL^−1^)	0.8 ± 0.0 ^a^	0.6 ± 0.0 ^b^	0.6 ± 0.0 ^b^	0.7 ± 0.0	0.6 ± 0.0	0.000	0.068	0.035
Urea(mg dL^−1^)	32.1 ± 1.4 ^a^	29.9 ± 1.0 ^ab^	26.6 ± 1.5 ^b^	30.0 ± 1.2	29.01 ± 1.1	0.012	0.506	0.005

^a–c^ Rows with different superscripts for same parameter are significantly different, DS = dry season, SR = short rainy season, LR = long rainy season, F = female, M= male, Total chol. = total cholesterol, gdL^−1^ = gram per deciliter, mg dL^−1^ = milligram per deciliter.

**Table 6:** Bodyweight of Fogera cattle calves under different seasons (mean ± SE).

Parameters	Season			Sex		*p*-Value		
	DS	SR	LR	F	M	Season	Sex	Season × Sex
IBW (kg)	49.0 ± 2.5 ^c^	63.5 ± 2.3 ^b^	64.8 ± 2.7 ^a^	59.2 ± 2.8	59.1 ± 1.8	0.0001	0.9688	0.0907
FBW (kg)	67.0 ± 2.8 ^c^	77.6 ± 2.5 ^b^	89.0 ± 2.4 ^a^	78.6 ± 2.7	77.2 ± 2.5	0.0001	0.6560	0.3800
TG (kg)	18.0 ± 2.1 ^b^	14.0 ± 2.2 ^c^	24.3 ± 2.3 ^a^	19.4 ± 1.8	18.1 ± 2.0	0.0038	0.6080	0.0127
ADG (g/d)	600.0 ± 0.07 ^b^	500.0 ± 0.1 ^c^	800.0 ± 0.07 ^a^	0.7 ± 0.1	0.6 ± 0.1	0.0036	0.6122	0.0126

^a–c^ Rows with different superscripts for same parameter are significantly different, DS = dry season, SR = short rainy season, LR = long rainy season, SEM= standard error mean, F = female, M = male, S = season. IBW (kg) = initial body weight in kilogram, FBW (kg) = final body weight in kilogram, TG (kg) = total gain in kilogram, ADG (g/d) = average daily gain in gram per day.

## Data Availability

This is not applicable to this article since no data sets were generated.
